# Mechanisms of Flexible Information Sharing through Noisy Oscillations

**DOI:** 10.3390/biology10080764

**Published:** 2021-08-10

**Authors:** Arthur S. Powanwe, Andre Longtin

**Affiliations:** 1 Department of Physics, University of Ottawa, 150 Louis Pasteur, Ottawa, ON K1N 6N5, Canada; alongtin@uottawa.ca; 2 Centre for Neural Dynamics, University of Ottawa, Ottawa, ON K1N 6N5, Canada; 3 Department of Cellular and Molecular Medicine, University of Ottawa, 451 Smyth Road, Ottawa, ON K1H 8M5, Canada

**Keywords:** noisy oscillations, mutual information, neural network

## Abstract

**Simple Summary:**

To properly interact with our environment, the brain must be able to identify external stimuli, process them, and make the right decisions all in a short time. This may involve several brain regions interacting together by sharing information birectionally via rhythmic activity. Such flexibility requires the functional connectivity between the areas to be dynamic, and a key question is the relevant parameter and operating regimes that make this possible in spite of fixed structural connectivity. Working towards this goal, we consider two coupled brain regions, each of which exhibits a noisy rhythm, a commonly observed type of neural activity. Such rhythms can be induced by the stochasticity in the neural circuitry, or be autonomously generated through nonlinearities and not necessitating noise. For these two types of rhythms, we computed the amount of information shared between the brain areas and the preferred direction(s) of sharing. We found that without the coupling delay, the flexibility needed by the brain to perform cognitive tasks requires the rhythms to be autogenerated rather than noise-induced. This is the case even with asymmetry or heterogeneity. This suggests that the importance of the dynamical regime has to be taken into account when modeling interacting neural rhythms from an information theoretical point of view.

**Abstract:**

Brain areas must be able to interact and share information in a time-varying, dynamic manner on a fast timescale. Such flexibility in information sharing has been linked to the synchronization of rhythm phases between areas. One definition of flexibility is the number of local maxima in the delayed mutual information curve between two connected areas. However, the precise relationship between phase synchronization and information sharing is not clear, nor is the flexibility in the face of the fixed structural connectivity and noise. Here, we consider two coupled oscillatory excitatory-inhibitory networks connected through zero-delay excitatory connections, each of which mimics a rhythmic brain area. We numerically compute phase-locking and delayed mutual information between the phases of excitatory local field potential (LFPs) of the two networks, which measures the shared information and its direction. The flexibility in information sharing is shown to depend on the dynamical origin of oscillations, and its properties in different regimes are found to persist in the presence of asymmetry in the connectivity as well as system heterogeneity. For coupled noise-induced rhythms (quasi-cycles), phase synchronization is robust even in the presence of asymmetry and heterogeneity. However, they do not show flexibility, in contrast to noise-perturbed rhythms (noisy limit cycles), which are shown here to exhibit two local information maxima, i.e., flexibility. For quasi-cycles, phase difference and information measures for the envelope-phase dynamics obtained from previous analytical work using the Stochastic Averaging Method (SAM) are found to be in good qualitative agreement with those obtained from the original dynamics. The relation between phase synchronization and communication patterns is not trivial, particularly in the noisy limit cycle regime. There, complex patterns of information sharing can be observed for a single value of the phase difference. The mechanisms reported here can be extended to I-I networks since their phase synchronizations are similar. Our results set the stage for investigating information sharing between several connected noisy rhythms in neural and other complex biological networks.

## 1. Introduction

The brain is a complex biological system composed of many subsystems. To perform important tasks such as perception, cognition and behaviour, its subsystems must coordinate information in a dynamic and flexible manner. Rhythmic activity is found in several brain areas such as the primary visual cortex [1,2] and observed in local field potentials and electroencephalograms. In particular, fast rhythms called gamma oscillations (30–100 Hz) are believed to play an important role in communication between areas [3,4,5]. One long-standing hypothesis for oscillatory communication in the brain assumes that it requires phase synchronization or coherence between interacting components [6,7,8]. According to this communication through the coherence (CTC) hypothesis, dynamic changes in neuronal synchronization should allow flexibility in communication in terms of directionality and timing. However, anatomical connections between brain areas are fixed which may, along with noise, impede flexible CTC. Thus, basic stochastic aspects of flexible communication with fixed connections are not well understood, and advancing knowledge in that direction is a prelude to a deeper view of CTC in the presence of connection strength variations due to synaptic and other plasticity mechanisms.

From a dynamic point of view, two distinct mechanisms can give rise to oscillations. One is the self-sustained periodic rhythm [9] with a constant amplitude, frequency, and regularly increasing phase. Such a “limit cycle” becomes a noise-perturbed limit cycle in the presence of weak noise. The other is the noise-induced rhythm (see [10] and references therein). Such a “quasi-cycle” requires noise to exist, otherwise any oscillatory behaviour stemming from an initial condition decays to and is replaced by a constant. With noise, the quasi-cycle amplitude fluctuates more strongly than for a limit cycle, as do the frequency and phase. In neuroscience, quasi-cycles are likely to include in vivo short epochs of synchrony called “bursts”, during which the amplitude of the collective rhythm is high [2,11,12]. Burst onset times and durations are random variables whose statistics can determine healthy vs. diseased brain states. The main motivation of our study is to advance our knowledge of how quasi-cycles can synchronize between areas and thus share information in spite of this burstiness, especially in comparison to noise-perturbed limit cycles.

The basic stochastic nature of quasi-cycles may limit their ability to synchronize with other brain areas displaying rhythms. Alternately, it may endow them with more flexibility compared to noisy limit cycles. We will see below that the issue of flexibility is a subtle one, even if the connection delays between two individually rhythmic subsystems are negligible as assumed in our work. In fact, the shape of the delayed mutual information function (dMI—defined below) between two gamma rhythm-generating subsystems, including the number, location and heights of its peaks, will be shown below to critically depend on the origin of the rhythms, the degree of heterogeneity of the networks and the asymmetry of their coupling.

The synchronization of gamma oscillations has been mostly studied using noise-perturbed limit cycles, for which an analysis in terms of phase synchronization is effective [13]. Phase synchronization of coupled noisy limit cycles has been intensively studied these last decades using simple models of interacting phases such as the Winfree and Kuramoto models [14,15,16] or more realistic biophysical mean-field [17,18] and spiking [19] models. The phase-locking of two noisy limit cycles is usually analyzed using the dynamics of their phase difference [20]. A mean phase difference of zero signifies In-phase locking (IP), while a mean of ±π indicates Anti-phase locking (AP). Between these two cases is out-of-phase locking (OPL) which can be seen in coupled limit cycles, but also in coupled quasi-cycles [21].

The sign of the mean phase difference value signals the leadership between the two oscillators. Outof-phase phase synchronization between two identical and symmetrically coupled limit cycle oscillators may occur due to spontaneous symmetry breaking beyond a certain critical parameter [19,22]. Which oscillator becomes the leader depends on the initial conditions, but the leadership can be dynamically reversed by a brief external pulse or by external noise, i.e., without altering the symmetry of the system [23]. Then, for a fixed anatomic or structural connectivity, the phase synchronization fluctuates [24], with possibly several peaks in the phase difference density. This suggests flexibility in information sharing by the oscillators as a steady state behaviour.

In contrast, phase synchronization has been much less studied for quasi-cycles in spite of the obvious relevance [2,11,12]. Only a few studies have addressed this issue [25,26]. Quasi-cycles can also exhibit robust phase synchronization. However, coupled quasi-cycles do not exhibit spontaneous symmetry breaking, and the presence of OPL relies on both coupling delay and noise [21]. That study shows that phase synchronization is nevertheless still present and dynamic despite the fixed anatomical connectivity. Moreover, such quasi-cycles can be accurately described by envelope-phase equations governed by a small set of meta-parameters. Thus, since quasi-cycles exhibit dynamic changes in phase differences, they should also show flexibility in information sharing. However, a coupling delay was essential for dynamic changes in phase differences. Here, we wish to investigate what happens without coupling delay but in more realistic cases where asymmetry and heterogeneity are considered.

Additionally, it is still not clear what is the precise relationship between phase difference dynamics and information flexibility. Can the pattern of information sharing or communication be inferred from the pattern of phase synchronization? Do noisy limit cycles differ in important ways from quasi-cycles in terms of flexible communication, and if so, what are the critical parameters and essential ingredients? In particular, how is the pattern of information sharing shaped by biophysically more realistic network features such as connection asymmetry or heterogeneity of the subsystems? These questions motivate our work. They are addressed using two subsystems, each exhibiting its own rhythm via the Pyramidal Interneuron Network Gamma (PING) mechanism. The two subsystems representing neighboring—and perhaps co-localized—brain areas are coupled through long-range excitatory connections. We study information sharing between these subsystems by computing the dMI between their phases [27,28]. The amplitudes of the rhythms are not explicitly analyzed in our study.

We first present the model, then the statistics used to quantify the phase and mutual information. Results are shown first for the quasi-cycles; these results are then confirmed by analyzing the phase differences and flexibility using a recently proposed envelope-phase description of the coupled quasi-cycle rhythms. Then follow our findings for the coupled noisy limit cycles. For each of these dynamical regimes, we investigate the effect of asymmetry in the anatomical coupling and heterogeneity in each population. The paper ends with a discussion and outlook onto future work. The results can be summarized as follows:

- Zero-delay-coupled quasi-cycles can exhibit robust phase-synchronization even in more realistic cases where asymmetry and heterogeneity are considered;
- The system of two coupled quasi-cycles oscillations does not show dynamic connectivity. The information is predominantly shared from one network to the other in the asymmetric inter-areal connectivity case and in the heterogeneous population case. Therefore it lacks flexibility in information sharing;
- When the system is in the noisy limit cycle regime, we may observe dynamic connectivity highlighted by the presence of two out-of-phase locking states for a single and fixed value of the inter-areal coupling. Information can then be shared from one network to the other and vice versa: there is a flexibility in information sharing. Such flexibility persists in the presence of asymmetry and heterogeneity but with some bias.

## 2. Methods

### 2.1. The Model

**Figure 1:**
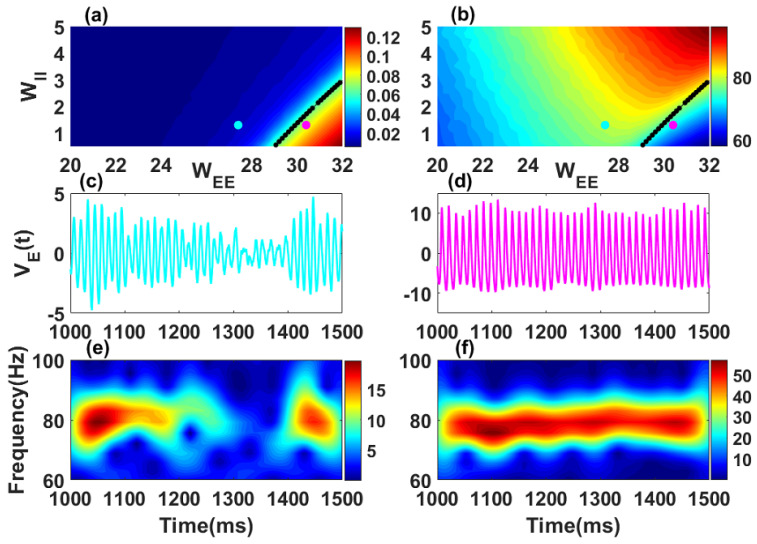
Oscillatory properties of a single stochastic Wilson–Cowan network. Synchronization level (**a**) and mean frequency (**b**) of the network are shown in color code in the recurrent inhibitory ($W_{II}$) vs. recurrent excitatory ($W_{EE}$) weight parameter subspace. Black dot curves in panels (**a**,**b**) define the Hopf boundary where the real parts of the rightmost complex conjugate eigenvalues $\lambda =-\nu \pm j\omega_{0}$ have ν=0. The quasi-cycle regime occurs for ν>0 and only in the presence of noise (see, e.g., the cyan point in (**a**) where $W_{EE}=27.4$); it has weak mean amplitude, i.e., weak synchronization. In the noisy limit cycle regime ν<0, limit cycles exist without noise, with amplitude and thus synchronization increasing as the parameters move away to the right of the boundary (representative point in magenta in (**a**) where $W_{EE}=30.4$). The amplitude is computed using a long simulation (80 s) of the excitatory activity E(t) in Equation (1) with $L_{EE}=0.0$ after transients. The two values of the mean frequency in (**b**) correspond to the high frequency gamma band (30–100 Hz). Fluctuations $V_{E}\left(t\right)$ are shown in (**c**) for the quasi-cycle regime and in (**d**) for the noisy limit cycle. Time-frequency representations of the excitatory fluctuations $V_{E}\left(t\right)$ are shown for the quasi-cycle in (**e**) and the noisy limit cycle in (**f**). Parameters are: $\alpha_{E}=0.1$, $\alpha_{I}=0.2$, $\beta_{E}=1.0$, $\beta_{I}=2.0$, $W_{EI}=26.3$, $W_{IE}=32$, $W_{II}=1.3$, $h_{E}=-3.8$, $h_{I}=-8.0$, $N_{E}=$ 80,000 and $N_{I}=$ 20,000 for all panels. For this figure and all other figures, numerical simulations were performed using the Euler-Maruyama scheme with a fixed integration step size of dt=0.05 ms.

**Figure 2:**
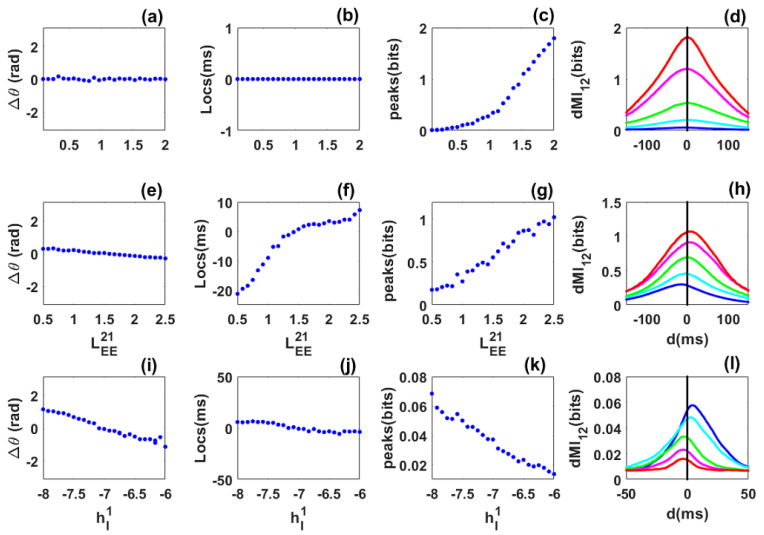
Phase locking and information sharing between two excitatorily coupled EI networks: Quasi-cycle regime. (**a**–**d**) **Symmetric case**. (**a**) Mean phase difference between the networks as the symmetric excitatory coupling ($L_{EE}^{12}=L_{EE}^{21}$) increases. (**b**) Location of the dMI peak vs. coupling strength. (**c**) Peak dMI value increases with coupling strength. (**d**) dMI vs. lag *d* with coupling strengths $L_{EE}^{21}=\left[0.48,0.86,1.24,1.62,2\right]$ for blue, cyan, green, magenta and red, respectively. (**e**–**h**) **Asymmetric case**. The coupling $L_{EE}^{12}=1.5$ is fixed. (**e**) Mean phase difference now increases from negative to positive. (**f**) Peak location changes from negative to positive, i.e., a change in direction of information sharing occurs. (**g**) Peak dMI value increases with coupling. (**h**) dMI curves with $L_{EE}^{21}=\left[0.9,1.3,1.7,2.1,2.5\right]$ for blue, cyan, green, magenta and red, respectively. (**i**–**l**) **Heterogeneous case**. (**i**) Mean phase difference between the networks as the inhibitory input to the first network $h_{I}^{1}$ varies from −8 to −6, while the inhibitory input to the second network $h_{I}^{2}=-7$ is fixed. (**j**) Peak location changes from negative to positive showing that there is a preferred direction of information sharing between the networks. (**k**) dMI peak value decreases as the inhibitory input to the first network increases. (**l**) dMI curves corresponding to $h_{I}^{1}=\left[-8,-7.6,-7.2,-6.8,-6.4\right]$ for blue, cyan, green, magenta and red, respectively. Other parameters are: $\alpha_{E}=0.1$, $\alpha_{I}=0.2$, $\beta_{E}=1$, $\beta_{I}=2$, $W_{EE}=27.4$, $W_{EI}=26.3$, $W_{IE}=32$, $W_{II}=1.3$, $h_{E}=-3.8$, $N_{E}=$ 80,000 and $N_{I}=$ 20,000 for all panels. For panels (**a**–**d**), $h_{I}^{1}=h_{I}^{2}=-8$ and for panels (**i**–**l**), $L_{EE}^{12}=L_{EE}^{21}=1$.

**Figure 3:**
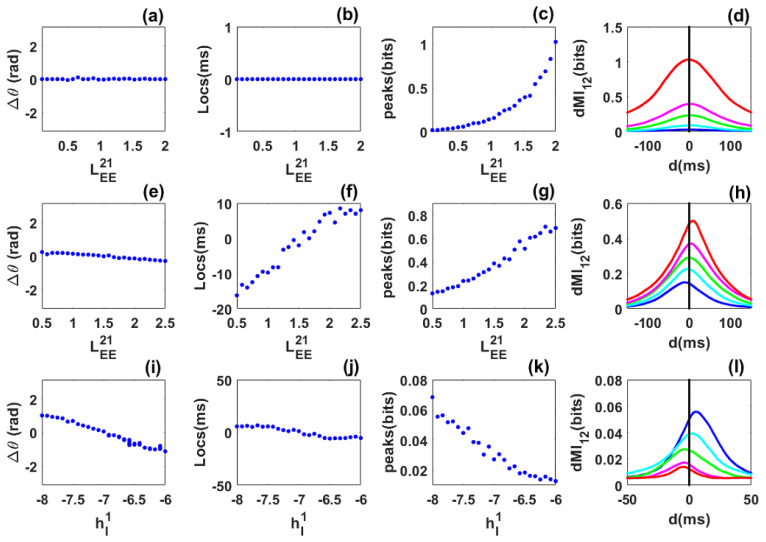
Phase locking and information sharing between networks coupled through long-range excitatory connections in the quasi-cycle regime: SAM analysis. (**a**–**d**) **Symmetric case**. (**a**) Phase difference between the networks as the symmetric long-range excitatory coupling ($L_{EE}^{12}=L_{EE}^{21}$) varies. The phase-difference value is always near zero. (**b**) dMI peak location is always at zero showing that there is no preferred direction of information sharing between the networks. (**c**) dMI peak value increases with coupling. (**d**) dMI curves for selected values of the long-range excitatory coupling. (**e**–**h**) **Asymmetric case**. (**e**) Phase difference between the networks as the long range excitatory coupling $L_{EE}^{21}$ varies while the coupling $L_{EE}^{12}=1.5$ is fixed. The phase-difference value increases from negative to positive. (**f**) Peak location changes from negative to positive: there is a preferred direction of information sharing between the networks. (**g**) Peak value increases with coupling. (**h**) dMI curves for selected values of the control parameter, showing changes in the location of the single peak. (**i**–**l**) **Heterogeneous case**. (**i**) Phase difference as the inhibitory input to the first network $h_{I}^{1}$ varies while the inhibitory input to the second network is fixed at $h_{I}^{2}=-7$. The phase-difference value decreases from positive to negative. (**j**) Change in the peak location from negative to positive, showing that there is a preferred direction of information sharing between the networks. (**k**) Peak value decreases as the inhibitory input to the first network increases. (**l**) dMI curves for selected values of the inhibitory input to the first network. Parameters are the same as in Figure 2.

**Figure 4:**
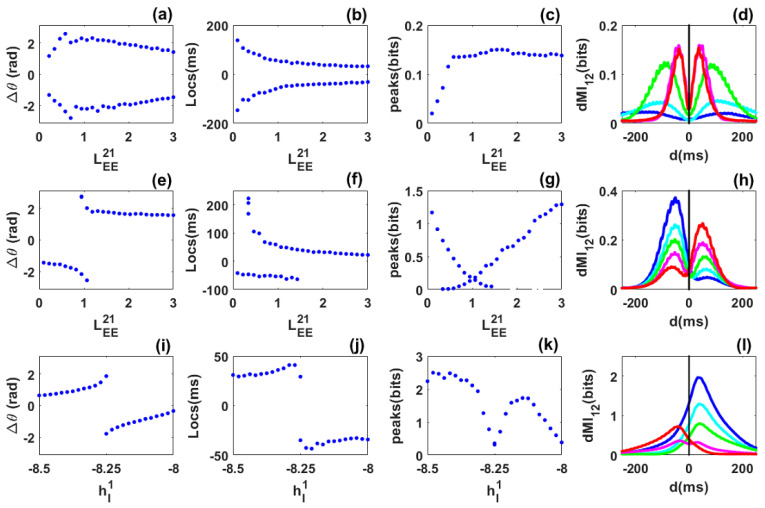
Phase locking and information sharing between networks coupled through long range excitatory connection in the limit cycle regime. (**a**–**d**) **Symmetric case**. (**a**) Phase difference between the networks as $L_{EE}^{12}=L_{EE}^{21}$ varies. The phase-difference distribution exhibits two symmetric preferred values. (**b**) Peak locations are symmetric, i.e., there are two equivalent preferred directions for information sharing between the networks. (**c**) Diagram of the peak values. (**d**) dMI curves for selected values $L_{EE}^{21}=\left[0.1,0.216,0.564,1.58,1.84\right]$ for blue, cyan, green, magenta and red, respectively. (**e**–**h**) **Asymmetric case**. (**e**) Phase difference as $L_{EE}^{21}$ varies while the coupling $L_{EE}^{12}=1.0$ is fixed. The phase-difference distribution exhibits two different values for the same coupling in a certain range. (**f**) Peak locations show bimodality for a certain range of the coupling. (**g**) dMI peak values. (**h**) dMI curves for $L_{EE}^{21}=\left[0.796,0.912,1.028,1.144,1.26\right]$ for blue, cyan, green, magenta and red, respectively. (**i**–**l**) **Heterogeneous case**. (**i**) Phase difference as the inhibitory input to the first network $h_{I}^{1}$ varies while the inhibitory input to the second network is fixed at $h_{I}^{2}=-8.25$. (**j**) The diagram of peak locations shows that there is a preferred direction of information sharing. (**k**) Diagram of the peak values. (**l**) dMI curves for selected values of the inhibitory input to the first network: $h_{I}^{1}=\left[-8.3,-8.28,-8.26,-8.24,-8.22\right]$ for blue, cyan, green, magenta and red, respectively.

We consider a system composed of two E-I networks [10] connected through long-range excitatory connections. A spike emitted by a neuron in the network *i* arrives instantaneously at a neuron of the network *j* with i,j∈{1,2}. We only consider the long-range excitatory connection between the E population of network *i* and the E population of network *j* denoted $L_{EE}^{ij}$. For each isolated network, the mean synaptic coupling from E cells to themselves, and from E cells to I cells, are given, respectively, by $W_{EE}$ and $W_{IE}$. Similarly, the mean synaptic coupling from I cells to themselves and from I cells to E cells are $W_{II}$ and $W_{EI}$. The E cell population receives a constant external stimulus $h_{E}$, while the I cell population is driven by the constant external current $h_{I}$. The complete description and numerical values of all the parameters are given in Table 1 and in the captions of Figure 1, Figure 2, Figure 3 and Figure 4. The dynamics of the E and I populations in each network are, respectively, given by:

(1) $$\begin{array}{ccc} \dot{E_{i}}\left(t\right) & = & -\alpha_{E}E_{i}\left(t\right)+\left(1-E_{i}\left(t\right)\right)\beta_{E}f\left(s_{E_{i}}\left(t\right)\right)+g_{1}\left(E_{i},I_{i}\right)\xi_{E_{i}}\left(t\right)\\ \dot{I_{i}}\left(t\right) & = & -\alpha_{I}I_{i}\left(t\right)+\left(1-I_{i}\left(t\right)\right)\beta_{I}f\left(s_{I_{i}}\left(t\right)\right)+g_{2}\left(E_{i},I_{i}\right)\xi_{I_{i}}\left(t\right)\\ s_{E_{i}}\left(t\right) & = & W_{EE}E_{i}\left(t\right)-W_{EI}I_{i}\left(t\right)+h_{E}+L_{EE}^{ij}E_{j}\left(t\right)\\ s_{I_{i}}\left(t\right) & = & W_{IE}E_{i}\left(t\right)-W_{II}I_{i}\left(t\right)+h_{I}^{i} \end{array}$$

with i,j={1,2}, i≠j, and where $s_{E_{i}}\left(t\right)$ and $s_{I_{i}}\left(t\right)$, i=1,2 are the total synaptic inputs to E and I populations in each network, respectively. The sigmoidal response of a neuron to its total input is given by f(x)=1/(1+exp(−x)), $\xi_{E_{i}}\left(t\right)$ and $\xi_{I_{i}}\left(t\right)$ are independent Gaussian white noises, and $g_{1}$ and $g_{2}$ are population-size-dependent multiplicative noise intensities defined by [10]:

(2) $$\begin{array}{ccc} g_{1}\left(E_{i},I_{i}\right) & = & \sqrt{\frac{\left(1-E_{i}\left(t\right)\right)\beta_{E}f\left(s_{E_{i}}\left(t\right)\right)+\alpha_{E}E_{i}\left(t\right)}{N_{E}}}\\ g_{2}\left(E_{i},I_{i}\right) & = & \sqrt{\frac{\left(1-I_{i}\left(t\right)\right)\beta_{I}f\left(s_{I_{i}}\left(t\right)\right)+\alpha_{I}I_{i}\left(t\right)}{N_{I}}}\;. \end{array}$$

The Gaussian white noises have the following properties:

$$\begin{array}{ccc} \langle \xi_{E_{i},I_{i}}\left(t\right)\rangle & = & 0,\;\langle \xi_{E_{i}}\left(t\right)\xi_{I_{i}}\left(t\right)\rangle =\langle \xi_{E_{i}}\left(t\right)\xi_{I_{j}}\left(t\right)\rangle =0\\ \langle \xi_{E_{i}}\left(t^{\prime }\right)\xi_{E_{i}}\left(t\right)\rangle & = & \langle \xi_{I_{i}}\left(t^{\prime }\right)\xi_{I_{i}}\left(t\right)\rangle =\delta \left(t-t^{\prime }\right)\;i,j=1,2\;. \end{array}$$

where the symbol $\langle .\rangle$ denotes the ensemble average. We focus on fluctuations around the fixed point defined as

(3) $$V_{E}^{i}=c_{E}\left(E_{i}\left(t\right)-E_{i0}\right)\;\;\;V_{I}^{i}=c_{I}\left(I_{i}\left(t\right)-I_{i0}\right)$$

where i=1,2, $c_{E}=\sqrt{N_{E}}$ and $c_{I}=\sqrt{N_{I}}$. Throughout our work, the population numbers are set to $N_{E}=$ 80,000 and $N_{I}=$ 20,000 in the typical approximate 4:1 ratio found in cortex. The effect of the noise is a function of the current state of the system. We choose the number of E and I neurons to be high, implying that the noise intensities are relatively weak. Moreover, they are kept fix for each set of parameters. Therefore we only investigate the effect of small noise intensities. Note that we are only integrating two equations of motion (E,I) for each oscillatory network; we are not modeling the activity of the individual 100,000 neurons. Additionally, we limited ourselves to two such networks coupled to one another.

### 2.2. Dynamics of a Single Stochastic Wilson–Cowan Network

The dynamics of a single isolated stochastic Wilson–Cowan network is given by Equations (1) and (2) without the index i,j and without the long-range excitatory couplings ($L_{EE}=0$). We focus on the parameter range where the deterministic dynamics ($g_{1}=g_{2}=0$) has complex conjugate eigenvalues $\lambda =-\nu \pm j\omega_{0}$ (Figure 1a), with imaginary part in the gamma band $\left(30\;\mathrm{Hz}<\omega_{0}/\left(2\pi \right)<100\;\mathrm{Hz}\right)$ (Figure 1b). Without noise $\left(g_{1}=0;\;g_{2}=0\right)$, the system admits a fixed point $\left(E_{0},I_{0}\right)$ which can be stable or unstable. When noise is included, the oscillatory behaviour of the system varies in the parameter space and depends on the stability of the fixed point.

***Synchronization level***: To quantify the ability of the model to generate sustained and coherent oscillations, we compute the “synchronization level” as the mean of the differences between the peak and the trough during each period over a very long simulation of the excitatory activity E(t) (see Figure 1a). In the two-population model, weak synchronization level refers to weak amplitude and less coherent oscillations whereas strong synchronization level correspond to high amplitude and coherent oscillations. Depending on the synchronization level, we can distinguish two different regimes. From [10], it is known that the amplitude, which is a measure of the mean firing rate across a network, is a reasonable proxy for the level of synchronization in an actual network of E and I cells (here one network is described only by two variables). However it is not a direct measure of synchronization, and in particular, this proxy will miss out on any synchronization that can occur at low firing rate levels [29].

***Quasi-cycle regime***: If ν>0 the fixed point is a stable focus. The synchronization level is weak as shown in the left part of the black curve (blue color) of Figure 1a. Oscillations are induced by noise, without which they would simply decay to the fixed point. The quantity of interest is therefore the fluctuation $V_{E}\left(t\right)=c_{E}\left(E\left(t\right)-E_{0}\right)$ of the excitatory activity around the fixed point. This regime is known in the literature as *quasi-cycle regime*. Note that we choose to work with only the excitatory variable E(t). However, the results obtained here are qualitatively similar with other quantities such as the sum of excitatory and inhibitory populations. The time series $V_{E}\left(t\right)$ of a representative point in the quasi-cycle regime (cyan dot in Figure 1a) is shown in Figure 1c and its corresponding time-frequency representation in Figure 1e. The characteristic bursting dynamics of the fluctuations in this regime can be seen from these panels as previously reported in other works [2,12].

***Noisy limit cycle regime***: For ν<0, the fixed point is unstable. Oscillations are generated independently of the presence of noise. The stochastic oscillations have a high synchronization level, and occur to the right side of the black curve (red color) in Figure 1a. This is the *noisy limit cycle regime*. The time series $V_{E}\left(t\right)$ for a representative point in the noisy limit cycle regime (magenta dot in Figure 1a,b) with its associated spectrogram are shown in Figure 1d,f. The oscillatory fluctuations in the noisy limit cycle regime are self-sustained with an almost constant frequency. The two oscillatory regimes are therefore distinct and may support different neural computations. The condition ν=0 (black curves in Figure 1a,b) corresponds to the transition between the two regimes known as the Hopf bifurcation.

***Mean frequency***; The parameters were chosen such that oscillation frequencies belong to the higher frequency gamma-band (30–100 Hz). To obtain the mean frequency, we first computed the autocorrelation function of the excitatory activity E(t). The autocorrelation is an even function with a maximum at the origin. The period of the signal thus corresponds to the value of the location of the second maximum at positive lags. The mean frequency is obtained by taking the inverse of this value. We used this approach to compute the mean frequency in Figure 1b.

### 2.3. Dynamics of Two Coupled Stochastic Wilson–Cowan Networks

In our work, we make sure that when two stochastic Wilson–Cowan networks operating in the quasi-cycle regime prior coupling are coupled through long-range excitatory connections ($L_{EE}^{ij}\neq 0$ in Equations (1) and (2)), the behaviour of the coupled system remains in the quasi-cycle regime. For coupled noisy limit cycle oscillators, the system also remains in the limit cycle regime for weak coupling. However, the stability of the deterministic fixed point $\left(E_{10},I_{10},E_{20},I_{20}\right)$ of the system is given by the rightmost eigenvalues ($\lambda =-\nu \pm j\omega_{0}$) of Equation (1). We did not consider the case where a network in the limit cycle regime (prior to coupling) is coupled to one in the quasi-cycle regime (prior to coupling).

### 2.4. Phase Locking

We are interested in the patterns of phase-locking and information sharing (i.e., communication) between phase signals of the reciprocally coupled networks. We extracted the phases using the Hilbert transform. For example, the analytic signal corresponding to $V_{E}\left(t\right)$ is $V_{E}\left(t\right)+jH\left[V_{E}\left(t\right)\right]$, with the Hilbert transform *H* defined as:

(4) $$H\left[x\right]=\frac{1}{\pi }P\int_{-\infty }^{\infty }\frac{x(\tau )}{t-\tau }d\tau$$

where *P* signifies the Cauchy principal value. The envelope of the stochastic signal is then $Env\left[V_{E}\right]=\sqrt{V_{E}^{2}+H^{2}\left[V_{E}\right]}$. Likewise, the phase angle of the analytic signal is defined as:

(5) $$\theta \left(t\right)=\arctan\left[H\left[V_{E}\right]/V_{E}\right].$$

***Phase difference distribution***: Phase locking can be analyzed by first computing the difference in the phases of the time series for each network. The phase difference is given by:

(6) $$\Delta \theta \left(t\right)=\theta_{1}\left(t\right)-\theta_{2}\left(t\right)\;,$$

where $\theta_{1}$ ($\theta_{2}$, respectively) is the phase of the excitatory population of the first network (the second network, respectively) extracted through the Hilbert transform. The most probable values, i.e., the location of the peaks Δθ of the distribution of Δθ(t), are the phase-difference values of interest.

***Phase cross-covariance function***: In the quasi-cycle regime, oscillations appear as short epochs of transient synchrony called bursts. Due to this transient nature, an alternative and more appropriate way to compute the phase-locking is to use the cross-covariance function between the phase signals defined as:

(7) $$C_{12}\left(s\right)={\langle \left(\theta_{1}\left(t\right)-\langle \theta_{1}\left(t\right)\rangle \right)\left(\theta_{2}\left(t+s\right)-\langle \theta_{2}\left(t\right)\rangle \right)\rangle }_{t}\;,$$

where the subscript *t* denotes a time average, and *s* varies from −T/2 and T/2 with *T* the mean period of the oscillation. The advantage of using the cross-covariance is that it takes into account the transient nature of the signals and therefore efficiently captures the correct phase-difference values. Using the cross-covariance function returns the most probable time-lag between the two signals. We convert this time-lag into a phase value using the mean period of the signal. The phase differences to which the system locks are then given by:

(8) $$\Delta \theta =2\pi \frac{s_{peak}}{T}$$

where $s_{peak}$ is the location (or locations) of the peak of the covariance function in the interval [−T/2,T/2]. The sign of the phase-difference value determines the leader-lag relationship between the two subsystems [23,26,30]. It is believed that if the phase difference is negative, the first network lags the second, and otherwise leads the second. The analysis of phase-difference values usually allows inferring the “effective connectivity” between the networks. Such connectivity can be different from the anatomical connectivity.

### 2.5. Delayed Mutual Information

Another way to infer the “effective connectivity” between the networks is to compute the delayed mutual information (dMI) using the phase signal of each network [27]:

(9) $$dMI_{1,2}\left(d\right)=\sum_{\theta_{1},\theta_{2}}P\left(\theta_{1}\left(t\right),\theta_{2}\left(t+d\right)\right)\log\left(\frac{P(\theta_{1}\left(t\right),\theta_{2}\left(t+d\right))}{P\left(\theta_{1}\left(t\right)\right)P\left(\theta_{2}\left(t+d\right)\right)}\right).$$

It measures the amount of information shared and transferred between the phases $\theta_{1}\left(t\right)$ of the first network and a time-shifted (by lag *d*) copy $\theta_{2}\left(t+d\right)$ of the second network as a function of the lag *d*, independent of how this information is encoded or decoded [27]. When the dMI curve has a single peak, or a most dominant peak, the sign of the location (i.e., time lag) of that peak gives the preferred direction of the information flow, and therefore the "effective connectivity" between the networks [27,28]. For example, if the location of a peak is positive, information flows preferentially from the first to the second network. Then, the system can be functionally interpreted as unidirectionally connected from the first to the second network even if there exists bidirectional anatomical connectivity. A peak at zero means that there is no preferred direction for information flow.

***Flexibility in information sharing: dynamic effective connectivity***: When the dMI curve exhibits a peak at both a positive and a negative location, the coupled networks reciprocally share information in both directions, in one direction at a time. The “effective connectivity” fluctuates in directionality from one epoch of time to another, and for the stochastic dynamics of interest here, these changes occur randomly, as described in [26]. Such behaviour is defined as *flexibility* in information sharing, or a dynamic effective connectivity, which is likely a necessary property for bi-directional communication between brain areas. In this picture, the more positive and negative peak locations there are, the more flexible the information sharing is. Note that this is fundamentally different from information sharing with a peak at zero, which does not imply the same flexibility.

***Amount of information shared***: The values of the peaks of the delayed mutual information describe the maximum amount of information shared between the networks. It also measures the level of phase synchronization between the networks. When there are several peaks at positive and negative locations, the system of coupled networks can still share information towards a dominant direction provided that one peak is higher than the other. The amplitudes of the dMI peaks will be studied in more depth elsewhere.

## 3. Results

### 3.1. Information Sharing between Quasi-Cycle Rhythms

***Symmetric case***: We first consider the symmetric case of two identical networks coupled with long-range excitatory connections $L_{EE}^{12}=L_{EE}^{21}$. The excitatory long-range connection $L_{EE}^{21}$ is taken as control parameter and varied from $L_{EE}^{21}=0.1$ to $L_{EE}^{21}=2$. The mean phase-difference values were always close to zero (Figure 2a) showing that the networks are in phase. The mean frequencies of the networks (not shown) increase with the coupling but remain equal. The location of the dMI peak is also at zero (Figure 2b). Thus there is no preferred direction for information sharing between the two networks, and no flexibility. The dMI peak increases with coupling (Figure 2c), a sign of enhanced synchronization and information sharing between the networks. Selected dMI curves are shown in /(Figure 2d), which confirm the results of (Figure 2b,c).

***Asymmetric-coupling case***: We move to two identical networks but which are asymmetrically coupled. The coupling $L_{EE}^{12}$ from the second to the first network is fixed at $L_{EE}^{12}=1.5$, while the coupling from the first to the second network $L_{EE}^{21}$ (taken as control parameter) is varied from $L_{EE}^{21}=0.5$ to $L_{EE}^{21}=2.5$. The phase-difference value decreases from positive ($L_{EE}^{21}<L_{EE}^{12}$) to negative ($L_{EE}^{21}>L_{EE}^{12}$) and is zero when $L_{EE}^{21}=L_{EE}^{12}$. When the coupling towards the first network is the greatest ($L_{EE}^{21}<L_{EE}^{12}$), the first network leads and the phase-difference values are positive; the reverse situation happens when $L_{EE}^{21}>L_{EE}^{12}$) (Figure 2e). The mean frequency of the first network is greater when $L_{EE}^{21}<L_{EE}^{12}$ and weaker when $L_{EE}^{21}>L_{EE}^{12}$. One could have expected that the network with a higher anatomical coupling to the other leads, but we observe the reverse situation.

The network with the higher frequency leads. The location of the dMI peak increases from negative to positive, meaning that information is preferentially shared from the second to the first network when the first network leads, and conversely when the second network leads (Figure 2f). The value of the dMI peak increases with the control parameter (Figure 2g). This is confirmed in Figure 2h. For a fixed structural connectivity, phase locking and information theory return a single value. Flexibility in information sharing is not present. Surprisingly, information sharing does not follow the phase relation in Figure 2e but the structural connectivity which is set by the values of $L_{EE}^{12}$ and $L_{EE}^{21}$. In other words information is not shared from the leader to the laggard as observed in previous studies [24,26,30] but from the network with a stronger structural or anatomical connectivity. This suggests that phase-relation does not necessary predict the direction of information flow as suggested [23,24,26]. Information may be shared from the laggard to the leader.

***Heterogeneous case***: Finally, we focus on the heterogeneous situation where the two networks are not identical. The inhibitory external input $h_{I}^{2}$ to the second network is fixed while the external input to the first is varied. The phase-difference values decrease from positive to negative. When the phase-difference values are positive ($h_{I}^{1}<-7$), the first network has a stronger power than the second, its mean frequency is also higher and it is the leader. For $h_{I}^{1}>-7$, we have the reverse situation (Figure 2i). The locations of the dMI peak also shifts from positive to negative according to the phase-difference values. Information is preferentially shared from the first to the second network when phase-difference values and locations are positive, and the reverse happens when these quantities are negative (Figure 2j). The dMI peak value decreases when the control parameter increases since the two networks become less synchronized and the amount of information shared decreases (Figure 2k). Information is preferentially shared from the leader to the laggard as predicted by previous studies [23,24,26,30]. However, flexibility in sharing is not seen since, for a fixed value of the structural connectivity, information can only be shared in one direction. These results are also seen in Figure 2l.

Thus for coupled quasi-cycles without propagation delay, phase synchronization is robust even in the presence of asymmetry and heterogeneity. However, communication is not flexible and the effective connectivity is not dynamic. This means that for a fixed structural connectivity or phase relation (values of Δθ), the dMI curves show a single peak. The network with the higher frequency leads. The pattern of information sharing between the networks cannot always be anticipated from the phase-locking dynamics as in previous studies [23,24,30], i.e., it is not always preferentially shared from the leader to the laggard but the reverse can happen according to the dominant structural connectivity. Additionally, the networks are “effectively” unidirectionally coupled in the asymmetric and heterogeneous cases. The phase difference relation (the phase-difference value) and the structural (values of the coupling) connectivity are unique for a given value of the control parameter and there is only one route for communication. The locations and peaks of the dMI depend on the biophysical parameters of the networks and their synchronization level.

### 3.2. Information Sharing between Quasi-Cycles: Envelope-Phase Decomposition Framework

In the quasi-cycle regime, it has been shown that dynamics of Local Field potentials (LFPs) could be written in the form:

(10) $$\begin{array}{cc} V_{E}^{1}\left(t\right) & =Z_{1}\left(t\right)\cos\left(\omega_{0}t+\phi_{1}\left(t\right)\right);\;V_{I}^{1}\left(t\right)=\alpha_{1}Z_{1}\left(t\right)\cos\left(\omega_{0}t+\phi_{1}\left(t\right)+\delta_{1}\right)\\ V_{E}^{2}\left(t\right) & =Z_{2}\left(t\right)\cos\left(\omega_{0}t+\phi_{2}\left(t\right)\right);\;V_{I}^{2}\left(t\right)=\alpha_{2}Z_{2}\left(t\right)\cos\left(\omega_{0}t+\phi_{2}\left(t\right)+\delta_{2}\right). \end{array}$$

The dynamics of the envelopes $Z_{i}\left(t\right)$ and the slow phases $\phi_{i}\left(t\right),\;i=1,2$ are obtained by performing the Stochastic Averaging Method (SAM) [12,21]. We can recover the fast phases $\theta_{i}\left(t\right)$ (which correspond to those extracted by the Hilbert transform) from the SAM phases $\phi_{i}\left(t\right)$ as:

(11) $$\begin{array}{c} \theta_{1}\left(t\right)=\omega_{0}t+\phi_{1}\left(t\right)\;\;\;\;\theta_{2}\left(t\right)=\omega_{0}t+\phi_{2}\left(t\right). \end{array}$$

This leads to the following envelope $Z_{i}\left(t\right)$ and phase $\theta_{i}\left(t\right)$ dynamics:

(12) $$\begin{array}{ccc} dZ_{1} & = & \left[-\lambda_{1}Z_{1}\left(t\right)+\frac{D_{1}}{2Z_{1}\left(t\right)}+\gamma_{1}\alpha_{1}C_{EE}^{12}\sin\left[\theta_{1}\left(t\right)-\theta_{2}\left(t\right)+\delta_{1}\right]Z_{2}\left(t\right)\right]dt+\sqrt{D_{1}}dW_{Z_{1}}\\ d\theta_{1} & = & \left[\omega_{1}+\gamma_{1}\alpha_{1}C_{EE}^{12}\cos\left[\theta_{1}\left(t\right)-\theta_{2}\left(t\right)+\delta_{1}\right]\frac{Z_{2}\left(t\right)}{Z_{1}\left(t\right)}\right]dt+\frac{\sqrt{D_{1}}}{Z_{1}\left(t\right)}dW_{\theta_{1}}\\ dZ_{2} & = & \left[-\lambda_{2}Z_{2}\left(t\right)+\frac{D_{2}}{2Z_{2}\left(t\right)}+\gamma_{2}\alpha_{2}C_{EE}^{21}\sin\left[\theta_{2}\left(t\right)-\theta_{1}\left(t\right)+\delta_{2}\right]Z_{1}\left(t\right)\right]dt+\sqrt{D_{2}}dW_{Z_{2}}\\ d\theta_{2} & = & \left[\omega_{2}+\gamma_{2}\alpha_{2}C_{EE}^{21}\cos\left[\theta_{2}\left(t\right)-\theta_{1}\left(t\right)+\delta_{2}\right]\frac{Z_{1}\left(t\right)}{Z_{2}\left(t\right)}\right]dt+\frac{\sqrt{D_{2}}}{Z_{2}\left(t\right)}dW_{\theta_{2}}.\; \end{array}$$

where the $dW_{k},k=Z_{i},\theta_{i}$ with i=1,2 are independent Brownian motions. The coefficients $\alpha_{i}$, $\delta_{i}$ with i=1,2 are, respectively, the amplitude ratio and phase difference between the inhibitory and excitatory fluctuations in each network. Their expressions can be found in [21]. The expressions of the effective coupling $C_{EE}^{ij}$ and the coefficients $\lambda_{i}$, $\gamma_{i}$, $D_{i}$ and $\omega_{i}$ are given by

$$\begin{array}{cc} C_{EE}^{ij} & =\left(1-E_{i0}\right)\beta_{E}f^{\prime }\left(s_{E_{i0}}\right)L_{EE}^{ij};\;\lambda_{i}=-\frac{A_{EE}^{i}+A_{II}^{i}}{2};\;\gamma_{i}=\frac{1}{2\alpha_{i}\sin\left(\delta_{i}\right)};\\ D_{i} & =\frac{\alpha_{i}^{2}\sigma_{E_{i}}^{2}+\sigma_{I_{i}}^{2}}{2{\left(\alpha_{i}\sin\left(\delta_{i}\right)\right)}^{2}};\;\omega_{i}=\gamma_{i}\left(\alpha_{i}\cos\left(\delta_{i}\right)\left(A_{EE}^{i}-A_{II}^{i}\right)+\alpha_{i}^{2}A_{EI}^{i}-A_{IE}^{i}\right) \end{array}$$

with the parameters

$$\begin{array}{ccc} A_{EE}^{i} & = & -\alpha_{E}-\beta_{E}f\left(s_{E_{i0}}\right)+\left(1-E_{i0}\right)\beta_{E}f^{\prime }\left(s_{E_{i0}}\right)W_{EE}\\ A_{EI}^{i} & = & -c_{EI}\left(1-E_{i0}\right)\beta_{E}f^{\prime }\left(s_{E_{i0}}\right)W_{EI}\\ A_{IE}^{i} & = & c_{IE}\left(1-I_{i0}\right)\beta_{I}f^{\prime }\left(s_{I_{i0}}\right)W_{IE}\\ A_{II}^{i} & = & -\alpha_{I}-\beta_{I}f\left(s_{I_{i0}}\right)-\left(1-I_{i0}\right)\beta_{I}f^{\prime }\left(s_{I_{i0}}\right)W_{II} \end{array}$$

and

$$\begin{array}{ccc} s_{E_{i0}} & = & W_{EE}E_{i0}-W_{EI}I_{i0}+h_{E}+L_{EE}^{ij}E_{j0}\\ s_{I_{i0}} & = & W_{IE}E_{i0}-W_{II}I_{i0}+h_{I}^{i}\\ \sigma_{E_{i}} & = & \sqrt{2\alpha_{E}E_{i0}};\;\sigma_{I_{i}}=\sqrt{2\alpha_{I}I_{i0}}\\ c_{EI} & = & \sqrt{N_{E}/N_{I}};\;c_{IE}=\sqrt{N_{I}/N_{E}}\;i,j=1,2. \end{array}$$

A complete and detailed review about the derivation of the envelope-phase dynamics Equation (12) can be found in [21]. We performed the same analysis as in the previous case where the phases were numerically extracted through the Hilbert transform. The goal is to assess the generality of the flexibility of communication through analytical models of envelope-phase dynamics Equation (12). We therefore compute the dMI using the analytical “Hilbert phases” obtained from simulations of Equation (12).

The results obtained for the symmetric, asymmetric, and heterogeneous cases in Figure 3 are qualitatively in very good agreement with those obtained numerically in Figure 2. However, we observe a quantitative difference in the number of bits shared between the networks. This may be due to the fact that our envelope-phase dynamics Equation (12) are based on the associated linear equations of the full SWC Equation (1), whereas the phase dynamics extracted through the Hilbert transform use the full nonlinear dynamics.

### 3.3. Information Sharing through Noisy Limit Cycles

To better understand the mechanism of information sharing reported for the case of noise-induced rhythms, we extend the previous analysis to the case of noisy limit-cycle oscillations. Parameters are such that, prior to coupling and without noise, each network lies in the limit cycle regime (Figure 1a, magenta dot), and when the long-range excitatory coupling is considered, the system of two coupled networks remains in the limit cycle regime for low value of the coupling. In contrast to previous work on phase locking and information sharing by noisy limit cycles [19,23,24], here we investigate more specifically the role of heterogeneity and coupling asymmetry in order to compare with the quasi-cycle case studied up to now. Another important difference is the fact that we did not include coupling delay in our modeling as in previous studies [19,22,24,31]. Coupling delay has been shown to induce dynamic out-of-phase locking and information sharing in identical and symmetrically coupled limit cycles oscillators through a mechanism called *Spontaneous symmetry breaking* [19,22,24]. It is therefore of interest to investigate if such dynamic phase locking and information sharing could be observe in the absence of coupling delay and if yes how such flexible information sharing behaves in the presence of asymmetry and heterogeneity.

***Symmetric case***: We consider identical networks, each of which exhibits a self-sustained rhythm in the absence of noise prior to coupling. We then symmetrically coupled them through long-range excitatory connection but without propagation delay. Unlike the case of quasi-cycle oscillations, we observe out-of-phase locking. In contrast to a previous study where propagation delay was included to explain out-of-phase locking between limit-cycles [19], the parameters chosen here are such that a delay is not necessary. However, the precise mechanism underlying this phenomenon (out-of-phase locking) is not identified here, as this question is beyond the scope of our present work. Additionally, noise is not critical for out-of-phase locking of limit cycles as it is for the quasi-cycles. Nevertheless, it causes the random reversals of leadership between the networks without any local intervention (such as stimulation).

The long-range excitatory coupling $L_{EE}^{21}=L_{EE}^{12}$ was again chosen as the control parameter. As it increased from $L_{EE}^{21}=0.1$ to $L_{EE}^{21}=3$, we observed that out-of-phase locking persisted (Figure 4a) but took on a range of phase difference values. The mean frequencies of the networks are equal (not shown). Interestingly, the diagram of the peak locations now shows two branches, i.e., for each value of the control parameter there exists two symmetric locations for the peaks as shown in Figure 4b. This already shows flexible communication between the two networks. The peak values are equal within statistical fluctuations. They first increase for weak coupling, and then stabilize for strong coupling (Figure 4c). The flexibility of communication is seen through the bimodality of selected dMI curves shown in Figure 4d. There is no need for propagation delay for flexible information sharing or dynamic effective connectivity. The precise mechanism behind the out-of-phase locking or dynamic effective connectivity is not well identified here. However, we found that such out-of-phase locking was different to the well-known spontaneous symmetry breaking as in previous studies [19,22,24]. A deeper investigation concerning this mechanism will be published elsewhere.

***Asymmetric-coupling case***: The coupling from the second to the first network is fixed $L_{EE}^{12}=1$, while the coupling from the first to the second network is the control parameter that varies from $L_{EE}^{21}=0.1$ to $L_{EE}^{21}=3$. For short values of this parameter ($L_{EE}^{21}<L_{EE}^{12}$), the second network leads and its mean frequency is higher. Then, over a short parameter range, the phase-difference diagram shows bimodality, i.e., when $L_{EE}^{21}$ is close to $L_{EE}^{12}$, the mean frequencies of the networks are equal. For the remaining range, the first network leads (i.e., for $L_{EE}^{21}>L_{EE}^{12}$—see Figure 4e) and its mean frequency is higher. The diagram of the dMI peak locations is also bimodal for a short range of the control parameter (Figure 4f). However, this range is larger than the bimodal range of the phase difference. This suggests that bimodality can be seen even for parameters where the phase-difference distribution is unimodal. Thus the phase-locking does not always reflect the pattern of communication.

The diagram of the peak values shows bimodality in agreement with the diagram of locations (Figure 4g). Bimodality corresponds to a low amount of information shared between the two networks. The flexibility reported in the locations and the values of the peaks of the dMI curves is highlighted in Figure 4h. Flexible information sharing persists when asymmetry is included between the networks but with a bias towards one network depending on the strength of the structural coupling. However, information is predominantly shared from the leader to the laggard according to the phase difference diagram.

***Heterogeneous case***: The inhibitory external input to the second network is fixed to $h_{I}^{2}=-8.25$ and the one applied to the first network is chosen as a control parameter and varied from $h_{I}^{1}=-8.5$ to $h_{I}^{1}=-8$. The long-range of excitatory coupling is symmetric. When $h_{I}^{1}<h_{I}^{2}$, network 2 has higher and stronger power than network 1. This implies that network 2 leads network 1 in phase as observed in Figure 4i, and the reverse occurs when $h_{I}^{1}>h_{I}^{2}$. There exists a value of the control parameter where phase-locking shows bimodality. This bimodality is observed in the diagram of locations in Figure 4j. Flexibility in communication is therefore present in the heterogeneous case. It seems then that increasing the long-range excitatory coupling leads to a broader range of flexibility. The values of the peaks decrease around the bimodality range as observed in previous cases (Figure 4k). Curves of dMI in Figure 4l reflect the phase-locking behaviour. The dominant direction of information sharing is from the leader to the laggard.

In summary, for coupled noisy limit cycles, delay is not critical for the appearance of dynamic out-of-phase locking and flexibility in information sharing. Such flexibility persists in the presence of asymmetry and heterogeneity. Information is predominantly shared from the leader to the laggard according to the phase-difference diagram. Noisy limit cycles therefore contrast with quasi-cycles since they allow flexibility which is not observed in quasi-cycles. This suggests that the functionality of the system of coupled networks depends on the dynamical regime.

## 4. Discussion

### 4.1. Summary of Results

We have studied information sharing between two networks, each of which exhibits its own rhythm through the PING mechanism; they are connected through long-range excitatory connections. Our principal goal was to identify critical parameters, regimes and mechanisms for flexible information sharing or dynamic effective connectivity in the absence of coupling delays. In particular, our study enables a direct qualitative comparison of information sharing between quasi-cycles and noisy limit cycles, which have both been advanced as candidate regimes for observed rhythms. We found that flexibility in communication depends on the dynamical origin of oscillations and, probably some critical biophysical parameters (not identified here). We note that a recent study has also showed the presence of symmetric out-of-phase locking states in coupled noisy limit cycles oscillators [32]. However, there was not a comparison with the quasi-cycle regime as we did here.

For identical and symmetrically coupled quasi-cycle oscillations, there is no flexibility in the absence of any delay. This likely follows from the fact that delays have been shown to be crucial for the presence of multiple out-of-phase locking (OPL) states, and without them, the range of phase differences between the networks is smaller. The same holds in the more realistic cases where asymmetry or heterogeneity is included in the model.

In contrast, for noisy limit cycle oscillations, flexibility is observed in the absence of coupling delay. Coupling delay is not necessary for dynamic changes in phase synchronization and flexible communication. However, the critical biophysical parameter responsible for dynamic out-of-phase locking observed with our parameter settings is not identified here. Flexible communication persists in more realistic situations where asymmetry or heterogeneity are included. This takes the form of two peaks in the dMI, and bistability in the phase difference.

Surprisingly, we found that the pattern of information sharing can not always be inferred from the phase relation. Even a single value of the phase difference can lead to a complex pattern of communication with two routes (see Figure 4e,f). This suggests that the relation between phase synchronization and communication is not trivial; preliminary results suggests that the situation is even more complex with delays, and this will be reported elsewhere in more detail. However, such differences between the pattern of phase locking (Figure 4e) and the mutual information (Figure 4f) are so far only the outcome of numerical experiments on phase locking and dMI. More theory is required to properly interpret the origin and significance of this interesting feature.

### 4.2. Limitations and Future Work

We also found that the relation between phase synchronization, structural coupling, frequency difference and information sharing depends on the specific coupling scenario and the dynamical regime of interest. More precisely, we showed that in the quasi-cycle regime, information could be shared from the laggard to the leader (in asymmetric coupling), in contrast with the noisy limit cycle regime where information was shared from the leader to the laggard. The relation between the phase difference and the frequency difference was similar in the quasi-cycle and noisy limit cycle regimes. In these cases, the network with the higher frequency was the leader, whereas the one with the lower frequency was the laggard. The fact that information could be shared from the phase laggard to the phase leader in quasi-cycles stands in clear contrast to previously known results on phase synchronization and information sharing between coupled limit cycle oscillators where the opposite was established [23,24,30]. Thus, further studies on phase synchronization of quasi-cycles are needed to further understand the dynamical origins of this result.

We used numerical simulations to compute phase synchronization and the dMI between phase signals. Phases were extracted through the Hilbert transform and from previously derived envelope-phase equations (SAM) for quasi-cycles. The results from the Hilbert transform and the SAM equations were qualitatively in good agreement. However, we observed a quantitative discrepancy between the amount of information shared and the exact locations of the peaks of the dMI curves. These discrepancies may be associated with the absence of nonlinear terms to obtain the SAM dynamics. In fact, SAM dynamics are derived from the linearized dynamics of the former nonlinear stochastic equations. Additional information could be contained in the nonlinear terms which were neglected to obtain the SAM dynamics to the order shown here. We also found (not shown) that the results obtained when using only the phase obtained directly from the SAM dynamics were identical to those obtained when the phases were converted to Hilbert phases. This suggests that all the information is contained in the stochastic part of the Hilbert phases.

Some results obtained here are in good agreement with previous results on flexible information transfer in noisy limit and quasi-cycles. These results allow us to gain a better insight into the mechanisms of flexible information sharing or dynamic effective connectivity between brain areas. However, complex biological, biochemical, genetic, mechanical, or chemical networks are often made up of more than two interacting subsystems. The results obtained here could be extended to such complex realistic networks. Furthermore, from a theoretical point of view, an extension of the envelope-phase dynamics to include nonlinearity and to cover limit cycle oscillations will be an important avenue for future analysis, along with the effect of external stimulation aimed at controlling the properties of the rhythms in isolation or under coupling.

## 5. Conclusions

We have addressed the question of flexible information sharing through neural oscillations. How can two coupled brain areas dynamically exchange information of a fast timescale provided that the structural or anatomic connectivity between them remains fixed? We tackled this question by considering oscillatory brain areas that independently exhibit rhythms in the fast gamma band (30–100 Hz) prior to being coupled. The two networks are connected through a bidirectional long-range excitatory connection but without conduction delay. For each of these two dynamical regimes, we computed the phase-locking states and the delayed mutual information between excitatory signals of the two brain areas. Our minimal criterion for flexible information sharing was two peaks in the delayed mutual information curve and phase difference distribution. We found that when the system of the two coupled brain areas was in the quasi-cycle regime, flexibility in information sharing was not observed. However, when the networks were in the noisy limit cycle regime, we observed flexibility in information sharing, i.e., sharing in both directions but not at the same time. Clearly, the effective connectivity was dynamic such that during a short epoch of time, only one network sent the information to the other which is passive, and the reverse situation happens during another epoch of time. Therefore, the ability of zero-delay coupled brain areas to dynamically exchange information while keeping the structural coupling fixed depends on the operating regime of the system. Including a coupling delay could induce more complex phase-locking patterns as well as several routes of information sharing even in the quasi-cycle regime, a possibility that further studies could establish.

## Figures and Tables

**Table 1:** List of parameters used in this work, along with their descriptions and their values.

Network Parameters	Description	Values
$W_{EE}$	recurrent excitation of each network	27.4/30.4
$W_{EI}$	feedback inhibition of each network	26.3
$W_{IE}$	feedback excitation of each network	32
$W_{II}$	recurrent inhibition of each network	1.3
$\alpha_{E}$	decay rate of excitatory neurons	0.1 ms^−1^
$\alpha_{I}$	decay rate of inhibitory neurons	0.2 ms^−1^
$\beta_{E}$	scaling coefficient of the E population response function	1
$\beta_{I}$	scaling coefficient of the I population response function	2
$N_{E}$	number of excitatory neurons	80,000
$N_{I}$	number of inhibitory neurons	20,000
$h_{E}$	external input to the E population of each network	−3.8
$h_{I}^{1}$	external input to the I population of network 1	see caption Figure 1, Figure 2, Figure 3 and Figure 4
$h_{I}^{2}$	external input to the I population of network 2	see caption Figure 1, Figure 2, Figure 3 and Figure 4
$L_{EE}^{12}$	long-range connection from $E_{2}$ to $E_{1}$	see caption Figure 2, Figure 3 and Figure 4
$L_{EE}^{21}$	long-range connection from $E_{1}$ to $E_{2}$	see caption Figure 2, Figure 3 and Figure 4

## Data Availability

Not applicable.
